# Congenital diaphragmatic hernia with concurrent aplasia of the pericardium in a foal

**DOI:** 10.1186/s12917-015-0623-2

**Published:** 2015-12-30

**Authors:** Alexandru-Flaviu Tăbăran, Andras Laszlo Nagy, Cornel Cătoi, Iancu Morar, Alexandra Tăbăran, Marian Mihaiu, Pompei Bolfa

**Affiliations:** Department of Veterinary Pathology, University of Agricultural Sciences and Veterinary Medicine, 3-5 Mănăştur Street, Cluj-Napoca, 400372 Romania; Department of Veterinary Toxicology, University of Agricultural Sciences and Veterinary Medicine, 3-5 Mănăştur Street, Cluj-Napoca, 400372 Romania; Department of Reproduction, Obstetrics and Veterinary Gynecology, University of Agricultural Sciences and Veterinary Medicine, 3-5 Mănăştur Street, Cluj-Napoca, 400372 Romania; Department of Animal Production and Food Safety, University of Agricultural Sciences and Veterinary Medicine, 3-5 Mănăştur Street, Cluj-Napoca, 400372 Romania; Department of Biomedical Sciences, Ross University School of Veterinary Medicine, Basseterre, St. Kitts, West Indies

**Keywords:** Congenital diaphragmatic hernia, Pericardial aplasia, Equine, Congenital defect, Malformation

## Abstract

**Background:**

In veterinary medicine congenital abnormalities of the diaphragm and pericardium are rare, idiopathic malformations, being reported mainly in dogs. This report documents an unusual case of developmental defects in a foal consisting of diaphragmatic hernia concurrent with pericardial aplasia.

**Case presentation:**

Following a normal delivery, a full term, female Friesian stillborn foal with the placenta was presented for necropsy. External morphological examination indicated a normally developed foal. At necropsy, a large oval defect (approximately 20 × 15 cm in size) was observed in the left-dorsal side of the diaphragm (left lumbocostal triangle). This defect allowed the intestinal loops, spleen and partially the liver to translocate into the thorax. The loops of the left ascending colon, including the pelvic flexure and partially the small intestine covered the cranial and dorsal posterior parts of the heart due to the complete absence of the left pericardium. The remaining pericardium presented as a white, semi-transparent strip, partially covering the right side of the heart. The left lung and the main bronchus were severely hypoplastic to approximately one-fifth the size of their right homologue. The intermediate part of the liver, containing mainly the enlarged quadrate lobe was translocated in the thorax, severely enlarged and showed marked fibrosis. Histologically in the herniated lobes we diagnosed hepatic chronic passive congestion, telangiectasia and medial hypertrophy of blood vessels.

**Conclusion:**

Concomitant malformation involving diaphragmatic hernia and pericardial aplasia in horses have not been previously reported. Moreover, this is the first case describing pericardial aplasia in horse.

## Background

Congenital diaphragmatic hernia (CDH) is a musculoskeletal defect defined by the presence of an orifice in the tendinous or muscular part of diaphragm, which allows the migration of abdominal viscera into the thorax [1, 2]. In horses congenital diaphragmatic hernias are rare developmental lesions associated with stillbirth [3], intermittent bowel obstruction [4] or rarely with newborn deadly colic [5]. Although less frequent in horses compared to post-traumatic hernias (diaphragmatic tear) [6, 7] from which sometimes are difficult to differentiate [3, 8], in several large-scale retrospective studies CDH is listed among the first ten important congenital malformations leading to abortion and stillbirth in foals [9, 10].

Congenital absence of the pericardium or pericardial aplasia represents a malformation which consists in the total absence (pericardial agenesis) or defects affecting portions of the pericardium [11, 12]. Idiopathic in nature, pericardial aplasia is diagnosed mainly during post-mortem investigations due to its usual asymptomatic evolution [13]. Although rarely reported in veterinary medicine, several cases of total pericardial aplasia were described in dogs [11, 13], mice [14], cattle [15] or orangutans [16].

Even if CDH is anatomically a simple defect, depending on the size of the diaphragmatic foramen, it is often observed associated with unilateral lung hypoplasia and with malposition of the abdominal organs (more frequently small intestine loops, and rarely, liver, spleen and stomach) in the thorax [6]. The number of organs involved and the severity of migration from abdominal to thoracic cavity are considered to be determined by the size of diaphragmatic defect, its location and the presence of the hernial sac which prevents the visceral displacement [4, 6]. Frequently reported in human pathology, CDH can be a part of a more complex congenital malformation involving other systems such as cardiovascular, genitourinary, neuronal and urogenital systems. In horses, CDH are rarely associated with concurrent skeletal malformation (as scoliosis, arthrogryposis and hypoplasia of the pelvic limb bones) [17, 18] or malformation affecting the urogenital system (uterus unicornis, unilateral renal and ureteral agenesis) as recently reported by Silva et al. [18]. Less frequently, CDH can be associated with cardiac defects. To the best of our knowledge, in horses only one reported case exists, described by Johnson et al*.* [19] in an Arabian foal, in which the CDH is associated with cardiac defects, in that case a ventricular septal defect.

Congenital diaphragmatic hernia with absence of the pericardium is an extremely rare combination, reported only in few cases of neonatal humans [20, 21]. This paper presents a rare case of diaphragmatic hernia associated with pericardial aplasia in a foal. We present the gross and microscopical description of the case, suggest and discuss possible etiologies and pathogenesis for each malformation and for their association, followed by a discussion from the perspective of comparative medicine.

## Case presentation

A full term female Friesian stillborn foal and most of it’s the placenta were presented for examination to the Pathology Department of the University of Agricultural Sciences and Veterinary Medicine Cluj-Napoca, Romania. The history of animal revealed that it was the third offspring of the same mating partners and the only stillbirth in the 2014 foaling season from a Friesian horse farm located in the central Transylvania. The foal resulted from normal, assisted labor, but after delivery, no vital signs were noticed. It was immediately submitted to cardiopulmonary resuscitation by the foaling attendant. Unfortunately, the procedure was not efficient. During pregnancy the mare had no history of vaccination, trauma, clinical disease or drug administration.

A complete post mortem examination was performed. For histology, the samples were fixed in 10 % neutral-buffered formalin and embedded in paraffin following the routine processing protocol. Four micrometers sections were stained using the hematoxylin and eosin (H&E) and Masson’s trichrome technique.

**Fig. 1:**
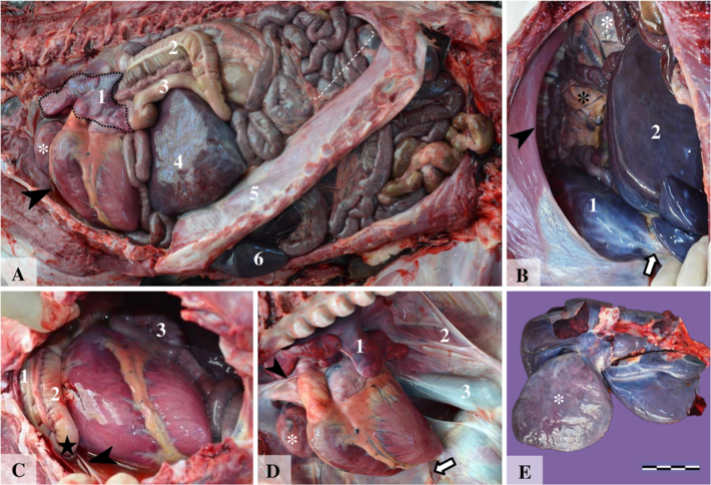
Foal, congenital pleuroperitoneal diaphragmatic hernia and left pericardium aplasia. Image **a**: Lateral view of the coelomic cavities after removal of the left abdominal and thoracic walls; hypoplastic left lung (1); the ventral (2) and the dorsal (3) loop of the left ascending colon; the thoracically translocated (4) and the abdominally located (5) parts of the liver; the diaphragm and area of the dorsal diaphragmatic defect (dotted accolade); the cranial lobe of the right lung which migrated cranial to the heart (white *). Image **b**: Caudal view of the large diaphragmatic defect with round and bold margins (arrow head) which allows the partial migration of liver and bowel (black *) from the abdomen into the thorax; fibrous aspect of the transdiaphragmatic migrated liver lobe (1) (extraabdominal region) connected by the abdominally located liver (2) by a stalk which contained dilated blood vessels and fibrous connective tissue (white arrow). The white * indicates the caudal pole of the left kidney. Image **c**: Heart and intestinal loops *in situ*. The dorsal (1) and the ventral (2) loop of the left ascending colon with the pelvic flexure (black star); small intestine loops (3) in contact with the heart; abundant serous fluid from the fused pleural and pericardial cavities and the partially formed pericardium (arrow head); Image **d**: The heart and the severely hypoplastic left lung (1) after removal of the intestinal loops; the right lung (2) and the caudal vena cava (3); the arrow indicates the remaining pericardium. Image **e**: Visceral surface of the liver. The parts of the liver which were translocated in the thoracic cavity (white*) were severely enlarged and exhibited diffuse fibrosis; bar = 10 cm

During necropsy, external examination indicated a normally developed female foal. The abdominal cavity contained approximately 500 ml of clear, yellowish fluid. A large ovoid opening located in the left dorsolateral part of the diaphragm was observed (Fig. 1a, and b), affecting the muscular and the tendinous components. Measuring approximately 20 × 15 cm, the foramen had a regular rounded, smooth shape, was firm, with no signs of hemorrhage (Fig. 1b). Through this defect the viscera from the abdominal cavity, including the small intestine loops, the cecum, ascending colon, spleen and the part of the liver (mainly the enlarged quadrate lobe) were displaced cranially in the thorax. No hernial sac was identified in the thorax. In the thorax, the two loops of the left ascending colon with the pelvic flexure and the jejunal loops were in direct contact with the cranial and dorso-caudal parts of the heart and large vessels (Fig. 1c). This was due to the lack of most of the left pericardium, the remaining structure presenting as a white, semi-transparent broad strip which extended from the basis of the heart to the sternum, along the right side the heart (Fig. 1d). The margins of the pericardial rudiment were uniform, slightly thickened and presented no signs of trauma. In the cranial part of the thorax due to an incomplete mediastinal septum, a part of the apical lobe of the right lung was displaced in the left hemithorax (Fig. 1d). Although covered by normal pleura, the left lung was severely compressed by the herniated organs. The hypoplastic lung tissue represented around one fifth of the size of the right lung.

The displaced liver was severely enlarged, with irregular margins, whitish and increased in consistency, suggestive of marked capsular fibrosis (Fig. 1e). The thoracically herniated liver was connected to the abdominal portion by a stalk which contained dilated blood vessels and fibrous connective tissue (Fig. 1b). The marked fibrotic change and the dilation of the blood vessels from the hilus of the thoracically herniated liver were due to the compression by the tendinous part of the diaphragm which formed the ventral part of the foramen. Other changes observed in other organs consisted of: varicose dilatation of the cardiac veins, thinned and fibrous aspect of the left kidney, on the cranial aspect. No morphological changes were observed in the submitted placenta.

**Fig. 2:**
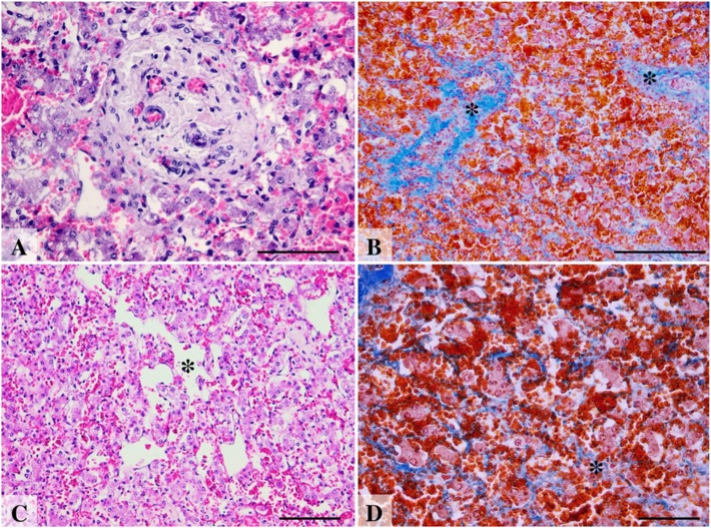
Histopathology changes in the translocated part of the liver. Image **a**: Chronic passive liver congestion and fibrosis in the herniated lobe. Portal area: fibrosis, medial hypertrophy of blood vessels and mild perisinusoidal fibrosis; H&E, scale bar = 50 μm; Image **b**: Liver, chronic passive liver congestion and fibrosis (blue) in the herniated lobe. Connective tissue deposition was observed in the proximity of the centrolobular vein and portal areas (*). MT, scale bar = 100 μm; Image **c**: Liver telangiectasia (*) in the herniated hepatic lobe marked by severe dilation of the sinusoidal capillaries and atrophy of the hepatocytes; H&E, scale bar = 100 μm Image **d**: Liver, prominent perisinusoidal fibrosis, congested sinusoid capillaries, atrophied hepatocytes and disrupted hepatic cords; MT, bar = 50 μm

Histopathological exam of the liver showed marked chronic, passive liver congestion and fibrosis with connective tissue formation mainly around the centrilobular vein and portal areas and multifocal areas of bridging fibrosis both between central and portal areas as well as between different portal areas (Fig. 2b, d). The changes in the portal area consisted of portal fibrosis and venous arterialization (Fig. 2a). Also areas of telangiectasia were noticed in both abdominal and herniated liver (Fig. 2c). In the lung, diffuse congestion and mildly thickened interstitium was noticed.

In terms of anatomical classification of congenital diaphragmatic hernias, the present case is best described as a dorsolateral (posterolateral) type of hernia (hernia through Bochdalek’s foramen or lumbocostal triangle). This form of CDH corresponds to Bochdalek type of hernia which represents 95 % of all cases of CDH in humans [22]. Moreover, in horses typical cases of CDH are located in the left-dorsal region of the diaphragm [6] as opposed to the retrosternal (Morgagni) [4] and post-traumatic hernias which frequently affect the ventral region of the right or left hemidiaphragm [3, 8]. Interestingly, in humans almost half (43 %) of the cases diagnosed with Bochdalek type of hernia also present additional structural defects [22] more frequently when compared to other types of CDH.

The etiology of congenital diaphragmatic hernia is unclear, even though multiple etiologies were proposed including: Vitamin A maternal deficiency [23], pesticides poisoning with diphenyl ether class, such as nitrofen [24] and cadmium chloride [25] or autosomal recessive inheritance (ex. chromosome 15q defects) [26]. Most cases of CDH remain idiopathic in nature [24].

The pathogenesis of CDH, although controversial, is related mainly to the malfunction of the retinoid signaling pathway (RSP) [24, 27, 28], caused by maternal deficiencies in vitamin A or toxic substances, teratogens that interfere with the normal function of RSP. It is also known that the herbicide nitrofen (2,4-dichloro-phenyl-p-nitrophenyl ether), today banned from both the European Union and the United States, administered in some period of gestation, produces CDH. Therefore, the use of this herbicide represents since the 1970’s the main experimental model in the CDH study [29]. In addition, other teratogens such as SB-210661, 4-biphenyl carboxylic acid (BPCA) and bisdiamine [N,N = −octamethylenebis (dichloroacetamide)], with similar structures with nitrofen were recently found to produce CDH and associated pulmonary hypoplasia and pulmonary vascular abnormalities [30]. All these teratogens inhibit the retinol dehydrogenase-2 (RALDH-2) enzyme, which plays an important role in the synthesis of retinoic acid [24]. This is supported also by the observation of the in utero nitrofen exposed animals which frequently develop aside from CDH also pulmonary and cardiac pathologies. Interestingly, even though less studied than CDH, in humans the pericardium aplasia is related to mutations in the same RALDH-2 gene [31]. The classical theory of the diaphragmatic embryogenesis demonstrates four primary embryonal structures which interact in the complete development of the diaphragm. These are: the septum transversum (the main primordium of the central tendon), the left and right pleuroperitoneal folds (the main origin of the muscular region), the mesentery of the esophagus (forms the crus of diaphragm), and the muscular segment of the latero-dorsal body walls (forms the peripheral regions of the diaphragm) [32]. These components have a different input in the diaphragm development. Recent theories have shown that the two pleuroperitoneal folds (PPF) are by far the major contributors to the diaphragm musculature, their incomplete fusion being the main event occurring in CDH [33]. Thus, in animals, the main event leading to diaphragmatic hernia has its origins in the destruction of the main mesenchyme primordia from the profound area of the pleuroperitoneal fold (PPF). The incomplete fusion of the pleuroperitoneal fold is based on the embryonal defect of the muscular component of the diaphragm and not an erroneous embryonal migration of the myocytes which would indicate a dysregulation of myogenesis per se which is often reported [30, 33]. Lung hypoplasia associated with congenital diaphragmatic hernias, as seen in our case, is frequently reported in both experimental [34] and spontaneous cases [32] of CDH. Due to this frequent association, a hypothesis that tried to explain the cause of CDH, suggested its origin in the primary pulmonary defect [35]. But this comorbid relation is best explained through the “Dual-hit hypothesis” formulated by Keijzer et al*.* [36]. This theory explains pulmonary hypoplasia by two consecutive developmental insults. The pulmonary hypoplasia in CDH initially originates from a common insult of diaphragmatic primordia (PPT), which perturbs the organogenesis of both structures (“first hit”), so as to further be involved in the compression resulted by the passing of the viscera from the abdominal cavity into the thoracic one through the diaphragmatic defect (“second hit”) [36]. Thus, CDH appears to be a primary developmental defect and not a secondary event resulting from ipsilateral lung hypoplasia [37].

The pericardial defect severity may range from total absence (pericardial agenesis) to defects affecting a portion of the pericardium [11] located in the left, right pericardium or diaphragmatic surface [38]. In dogs, like in humans, the defects affecting the left side of the pericardium are more frequently observed [11, 13]. Pericardial aplasia represents the partial defect, whereas agenesis the total congenital defect of the pericardium, which, based on anatomical classification are divided in total agenesis or unilateral agenesis of the left and right pericardium [39]. Sometimes the diaphragm and caudal pericardium share a common defect resulting in a peritoneopericardial hernia, a distinct form of diaphragmatic hernia previously described also in horses [40]. Congenital absence of the left pericardium is believed to appear in early embryogenesis due to premature atrophy of the left duct of Cuvier (left common cardinal vein). This atrophy is believed to be responsible afterwards of insufficient irrigation of the left pleuropericardial membrane, arrested development and defective fusion of pericardial primordia [41]. However, other theories which try to explain the origins of the pericardial defect by the errors of the lung bud migration [42] or abnormal enlargement of the heart [43] have been proposed.

Pericardial aplasia evolves asymptomatically, most often being an incidental finding during necropsy or diagnostic imaging [41]. Rarely, especially when the congenital partial defect is partial, the pericardial opening permits the herniation of the atrial appendage followed by strangulation [44]. The location of the defect in the left pericardium, which is by far the most frequent form of pericardial malformation in both humans and animals [13, 45], has been explained mainly by the embryologic normal “rotation of the heart” which determines a greater traction on the left side of the pericardium [45], and by the greater trauma to the left pulmonary ridge due to the asymmetry of the liver and its rotation during embryogenesis [46].

From a clinical perspective, in human cases of pericardium aplasia, the most frequently noted symptom was chest pain [47]. This was present in approximately one fourth of cases [48]. Its origin hypothetically was associated with the rotation of the heart into the left chest [47] due to the absence of the support role of the pericardium and increased tension on the anchoring structures of the heart [41]. Additionally, pectus excavatum [48], systolic heart murmurs on the pericardial defect [39, 49], sinus bradycardia [47] and dyspnea [50, 51] are occasionally observed. Although rarely reported, fatal syncope represents the most severe consequence of pericardial defect [52, 53]. The proposed mechanisms responsible for syncope are linked with neural vagal reflex followed by mediated bradycardia and hypotension [52] secondary to cardiac herniation and strangulation [53]. Additionally to this mechanism, severe hemodynamic compromise due to myocardial compressive ischemia is proposed [11].

Experimentally induced pericardial defect in 65 dogs was not associated with typical clinical signs since no impaired cardiac function or cardiac dilatation [54] were noticed. Similar experiments carried in cats by Carleton et al*.* [55] show no differences in heart-rate and blood pressure between the group with induced pericardial defect and cats used as control. Interestingly, following long term radiologic assessment, cardiac dilatation was noticed in 12 out of the 13 pericardectomised cats. Hypothetically this cardiac dilatation was attributed to the absence of limiting effect of the pericardium which normally restrains the heart [55]. In humans and dogs, spontaneous cases of pericardial defects are rarely associated which cardiac deformities [11, 13, 39, 49].

Recently Chapel et al*.* [11] described a case of a dog with syncope secondary to left auricle herniation through a congenital pericardial defect. The associated clinical signs were repetitive syncope episodes, coughing, tachycardia (atrial), heart murmurs (left apical and right parasternal holosystolic), irregular arterial pulses. Radiographically, generalized cardiomegaly (mild) with severe left auricular enlargement was recorded.

Interestingly, in humans, CDH and pericardial aplasia were also associated with liver heterotopia in three cases [20]. These defects were hypothetically linked to a common cause represented by the initial defect of the phrenic nerve, which is involved in the embryogenesis of both structures.

## Conclusions

This case of congenital diaphragmatic hernia with concurrent aplasia of the pericardium in a Friesian foal represents the first report of this association of congenital malformations in horses, and also the first description of pericardial aplasia in this species. Considering that in other species, such as dogs, this pathology was identified in several individuals, in horses pericardial aplasia can be an overlooked diagnosis, urging a better examination of this structure especially in the context of congenital diaphragmatic hernia. This is supported by the statement of Sir William Osler regarding the peculiarity of pericardial diseases: *“Probably no serious disease is so frequently overlooked by the practitioner”* [56].

## Abbreviations

CDH: Congenital diaphragmatic hernia
H&E: Hematoxylin-eosin
MT: Masson’s trichrome stain
RALDH-2: Retinaldehyde dehydrogenase 2
BPCA: 4-biphenyl carboxylic acid
SB-210661: S)-N-hydroxy-N-[2,3-dihydro-6-(2,6-difluorophenylmethoxy)-3-benzo furanyl]urea
RSP: Retinoid signaling pathway
